# Lutein is needed for efficient chlorophyll triplet quenching in the major LHCII antenna complex of higher plants and effective photoprotection *in vivo *under strong light

**DOI:** 10.1186/1471-2229-6-32

**Published:** 2006-12-27

**Authors:** Luca Dall'Osto, Chiara Lico, Jean Alric, Giovanni Giuliano, Michel Havaux, Roberto Bassi

**Affiliations:** 1 Dipartimento Scientifico e Tecnologico, Università di Verona, Strada Le Grazie 15, I-37134 Verona, Italy; 2 Ente per le Nuove tecnologie, l'Energia e l'Ambiente (ENEA), Unità Biotecnologie, Centro Ricerche Casaccia, C.P. 2400, Roma 00100, Italy; 3 CEA/Cadarache, DSV, DEVM, Laboratoire d'Ecophysiologie de la Photosynthèse, UMR 6191 CEA-CNRS-Aix Marseille II, F-13108 Saint-Paul-lez-Durance, France; 4 Laboratoire de Génétique et Biophysique des Plantes (LGBP), Département d'Ecophysiologie Végétale et Microbiologie – UMR 163 CEA-CNRS Université de la Méditerranée Aix-Marseille II, 163 Avenue de Luminy, Marseille, France; 5 Institut de Biologie Physico-Chimique (IBPC), rue Pierre et Marie Curie 13, Paris, France

## Abstract

**Background:**

Lutein is the most abundant xanthophyll in the photosynthetic apparatus of higher plants. It binds to site L1 of all Lhc proteins, whose occupancy is indispensable for protein folding and quenching chlorophyll triplets. Thus, the lack of a visible phenotype in mutants lacking lutein has been surprising.

**Results:**

We have re-assessed the *lut2.1 *phenotypes through biochemical and spectroscopic methods. Lhc proteins from the *lut2.1 *mutant compensate the lack of lutein by binding violaxanthin in sites L1 and L2. This substitution reduces the capacity for regulatory mechanisms such as NPQ, reduces antenna size, induces the compensatory synthesis of Antheraxanthin + Zeaxanthin, and prevents the trimerization of LHCII complexes. *In vitro *reconstitution shows that the lack of lutein *per se *is sufficient to prevent trimerization. *lut2.1 *showed a reduced capacity for state I – state II transitions, a selective degradation of Lhcb1 and 2, and a higher level of photodamage in high light and/or low temperature, suggesting that violaxanthin cannot fully restore chlorophyll triplet quenching. *In vitro *photobleaching experiments and time-resolved spectroscopy of carotenoid triplet formation confirmed this hypothesis. The *npq1lut2.1 *double mutant, lacking both zeaxanthin and lutein, is highly susceptible to light stress.

**Conclusion:**

Lutein has the specific property of quenching harmful ^3^Chl* by binding at site L1 of the major LHCII complex and of other Lhc proteins of plants, thus preventing ROS formation. Substitution of lutein by violaxanthin decreases the efficiency of ^3^Chl* quenching and causes higher ROS yield. The phenotype of *lut2.1 *mutant in low light is weak only because rescuing mechanisms of photoprotection, namely zeaxanthin synthesis, compensate for the ROS production. We conclude that zeaxanthin is effective in photoprotection of plants lacking lutein due to the multiple effects of zeaxanthin in photoprotection, including ROS scavenging and direct quenching of Chl fluorescence by binding to the L2 allosteric site of Lhc proteins.

## Background

The pigment composition of the photosynthetic apparatus of higher plants is extremely well conserved: chloroplast-encoded photosynthetic reaction center complexes bind β-carotene and chlorophyll *a*, while nuclear-encoded light harvesting proteins bind Chl *a*, chlorophyll *b *and the three xanthophylls lutein, violaxanthin and neoxanthin. In addition, plants exposed to excess light conditions synthesize antheraxanthin and zeaxanthin by a two step de-epoxidation of the existing violaxanthin [1]. β-carotene is also bound to the light harvesting complex of Photosystem I [2]. The conservation of carotenoid composition and distribution across a range of plant *taxa *suggests that each xanthophyll species serves a specific role. However, the reason for the co-existence of different xanthophyll species is not completely clear. In fact, all of the above-mentioned xanthophylls possess similar absorption characteristics in the visible region of the spectrum and are capable of quenching harmful chlorophyll triplets and reactive oxygen species produced during oxygenic photosynthesis [3]. Also, the energy level of the S1 state of different xanthophylls, which is critical for energy transfer from chlorophyll, is very similar both in solution and when bound to Lhc proteins [4,5]. Although a small fraction of xanthophylls is likely to be free into the thylakoid lipids, where they catalyze ROS scavenging and reduce lipid peroxidation [6,7], xanthophylls are mainly bound to the Lhc proteins of both PSI and PSII [8]. Recent work, using both recombinant proteins and carotenoid biosynthesis mutants, has suggested that the function of individual xanthophyll species can be understood within the framework of their binding to proteins of the Lhc family [9]. It was shown that the competitive binding of violaxanthin and zeaxanthin to the allosteric site L2 of Lhc proteins controlled the transitions between two conformations with respectively long and short fluorescence lifetime. This change is assumed to contribute to the regulation of light harvesting efficiency and of dissipation of excess light energy (reviewed in [10]).

Lutein is the most abundant carotenoid in the higher plant photosynthetic apparatus and the only ligand for site L1 in Lhc proteins, whose occupancy is essential for protein folding and the quenching of ^3^Chl* [9]. Early studies reported isolation of viable lutein-deficient mutants, showing no visible phenotype in laboratory conditions [11]). Later studies have shown that the *lut2 *mutant has alterations in NPQ kinetics, antenna size, and reduced LHCII trimer stability [12]. However, none of these studies reported an "*in vivo*" phenotype corresponding to the observed biochemical lesions and could suggest a specific functional role for lutein wth respect to other xanthophyll species but for a recent report of decreased growth and Fv/Fm upon stress in *lut2 *[13]. In this manuscript we report on the function of lutein in photosynthesis, through the isolation of a knock-out ε-cyclase mutant of *Arabidopsis thaliana*, *lut2.1*, and its characterization through biochemical and physiological methods. Detailed analysis in vivo and purified xanthophyll binding proteins allows individuate specific functional phenotypes, which are consistent with lutein being more efficient in chlorophyll triplet quenching than violaxanthin and suggesting that each xanthophyll species has a specific effect in chloroplast photoprotection.

## Results

### Pigment composition and photosynthetic functions

In agreement with previous results on *lut2 *mutant [14]*, lut2.1 *plants showed similar organ size compared to WT plants, but a slightly lower Chl content per fresh weight and leaf surface. When analyzed for their pigment composition [see Additional file 1] it appeared that the Chl *a/b *ratio was higher in *lut2.1 *with respect to WT as was the Chl/Car ratio. Lutein was completely absent from the mutant; a strong compensatory increase of violaxanthin was observed. WT dark-adapted plants did not contain any antheraxanthin or zeaxanthin which were, instead, found in *lut2.1 *leaves to low, but detectable amounts [14]. When exposed to strong light for 20 min, *lut2.1 *plants accumulated A+Z to levels approx 3 times higher than WT. In agreement with previous results [14], the quantum yield of PSII photochemistry (F_v_/F_m _chlorophyll fluorescence ratio) was not significantly different in *lut2.1 *with respect to WT. However, we found that the fluorescence quantum yield of Chl in dark-adapted plants was always lower in *lut2.1 *with respect to WT [see Additional file 2]. This observation suggests that some kind of constitutive thermal dissipation mechanism, resulting in the quenching of chlorophyll fluorescence, is activated in *lut2.1 *chloroplasts. According to [11], NPQ was higher in WT with respect to *lut2.1 *leaves [see Additional file 6]. The two genotypes differ for the initial rate of qE, which is much slower in *lut2.1*. The PSII antenna size was determined by measuring the half time in the rise of chlorophyll fluorescence in the presence of the photosynthetic electron transport inhibitor DCMU [15]. The half time was 65 ms in WT *vs*. 81 ms in *lut2.1*, suggesting that the functional antenna size was 20% smaller in the mutant [see Additional file 2]. These results support suggestions by Lokstein *et al*. [12] based on different methods.

### State I- State II transitions are impaired in lut2.1

The antenna sizes of PSI and PSII adapt to light quality by phosphorylating LHCII. Upon phosphorylation, this complex is disconnected from the PSII reaction center and diffuses to PSI complexes, where it increases light harvesting and electron transport capacity. This mechanism has been called state transition (see [16] for a review). We assayed the capacity for performing State I – State II transitions by measuring the increase in oxygen evolution when a far red light was superimposed to a background of blue-green light (Emerson effect). The state transition phenomenon was clearly visible in WT, with the Emerson effect being low in state II (ca. 5.5%, indicating an almost even distribution of the blue-green light energy between PSI and PSII) and high in state I (ca. 30%, indicating a strong imbalance in light energy in favor of PSII). In *lut2.1*, the change in the Emerson effect was very small, indicating that the capacity for change in antenna size of PSI through state I – state II transitions was severely impaired (Table 1). To our knowledge, this is the first evidence for a specific need of lutein in the mechanism of state transitions.

### Supramolecular organization of pigment binding complexes

**Figure 1:**
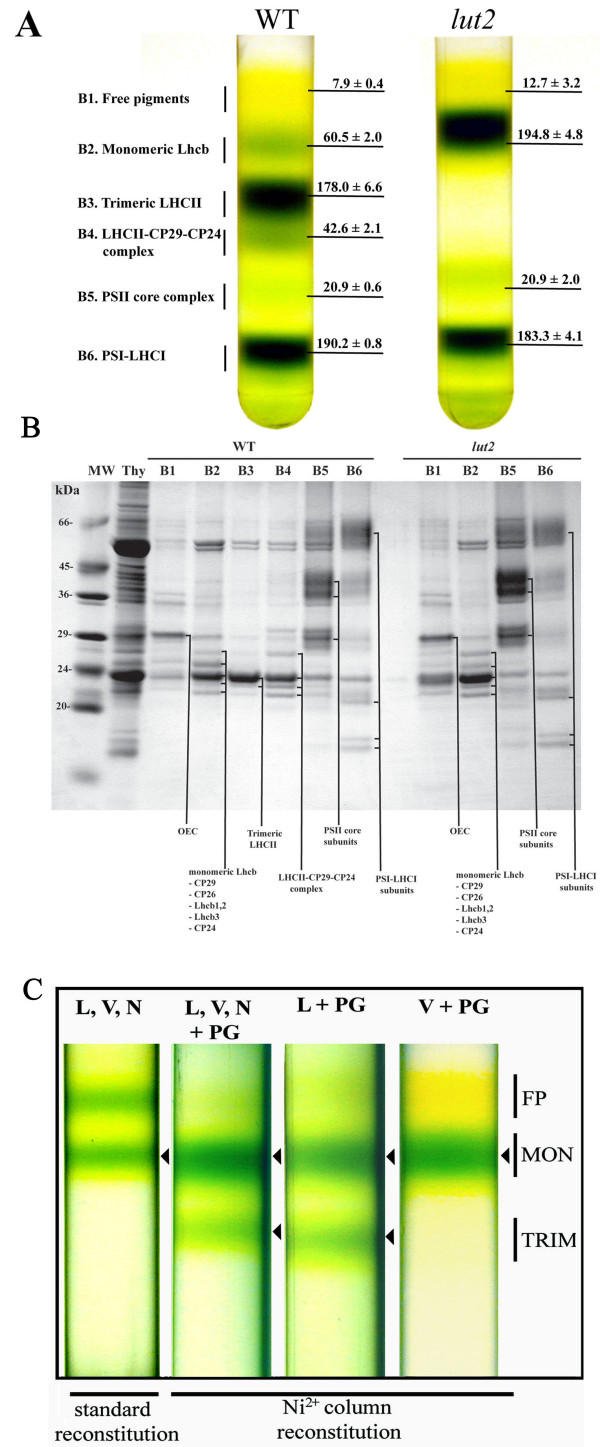
**A. Sucrose density gradient profiles of WT and *lut2.1 *solubilized thylakoids**. Thylakoid membranes from WT and *lut2.1 *plants were solubilized with α-DM and loaded on sucrose gradient; for each gradient, fractions harvested (*left*) and chlorophyll distribution (% of total Chl loaded) in the gradient along gradients (*right*) are indicated. Chlorophyll levels of each band were normalized to the Chl content of WT band 5. Data are expressed as mean ± SD, n = 3. **B. Gel electrophoresis of sucrose gradient fractions**. Tris-Tricine SDS-PAGE analyses of gradient bands from Figure 1A. Main protein components of each fraction are indicated. Figure abbreviations: B, band; Thy, thylakoids; MW, molecular weight marker. **C. Trimerization behavior of recombinant LHCII proteins**. LHCII were reconstituted *in vitro *with different xanthophyll species and trimerization of monomeric subunits was allowed by adding PG, a lipid factor essential for trimerization [67]. LHCII containing a mix of xanthophylls (L,V,N) or only lutein (L) produced trimers, while violaxanthin-binding complexes (V) did not produce trimers. See Experimental Procedures for details. FP, free pigments; MON, monomeric subunits; TRIM, trimeric complexes.

Reduced stability of LHCII trimers has been previously reported in the *lut2 *mutant [12]. Such phenotype could be, in principle, due to the altered pigment composition, or to altered protein composition of the complexes, or both. Thus, we decided to further these observations using sucrose density gradient fractionation of solubilized thylakoids, followed by SDS-PAGE of the fractions, and HPLC analysis of the pigment content of the fractions. The results of the fractionation are shown in Figure 1A. Five bands are visualized in the WT: Band 1 is yellow and contains free carotenoid pigments; band 2 contains the minor antenna complexes CP24, CP29 and CP26, and LHCII monomers; band 3 contains LHCII trimers; band 4 contains the LHCII-CP29-CP24 complex; band 5 contains the PSII core complex; and band 6 the PSI-LHCI complex. Mutant thylakoid membranes show the complete absence of band 3 (trimeric LHCII), while band 2 (monomeric LHCII) is much more represented than in WT. Upon normalization to the Chl content of the PSII core complex band, the Chl content associated to Lhc proteins in band 2+3 is lower in *lut2.1 *by approx. 10%, in agreement with the smaller functional PSII antenna size indicated by our fluorescence measurements and a previous report [12], while that associated to the PSI-LHCI complex is unchanged. SDS-PAGE analyses show that band 2 from *lut2.1 *contain the same polypeptides as the corresponding band from WT, although the relative amount of the Lhcb1-3 polypeptides, components of LHCII, is increased (Figure 1B). Overall, the data confirm that LHCII is present in the *lut2.1 *mutant but its aggregation state is monomeric rather than trimeric [12].

HPLC analyses of bands 2 and 3 (Table 2) indicate that V, A and Z are associated to the Lhcb proteins in band 2 of *lut2.1*, while in WT only V, N and L are found in bands 2 and 3.

We then asked if the lack of lutein and its substitution by violaxanthin in Lhc proteins, *per se*, was the actual reason for LHCII monomerization in *lut2.1*. In order to verify this point, we used recombinant Lhcb1 protein, overexpressed in bacteria, for reconstitution with different xanthophyll species plus Chl *a *and Chl *b*. Refolded proteins were then separated from free pigment by Ni^2+ ^column chromatography and fractionated by sucrose gradient ultracentrifugation in order to resolve different aggregation states. The results (Figure 1C) indicate that Lhcb1 reconstituted with a mix containing all pigments, as well as the complex with lutein only, did produce trimers. Conversely, if violaxanthin was supplied in the absence of lutein, a violaxanthin-binding complex was obtained which did not produce trimers. For the first time, our measurements show that the binding of lutein *per se *is sufficient for LHCII trimerization, and that violaxanthin cannot substitute for lutein in this function.

Lutein binds to specific sites within LHCII complexes [17], termed sites L1 and L2, while neoxanthin binds to site N1 and V+A+Z to the external site V1 [18]. Different binding sites provide slightly different protein environments, which are reflected in different shifts of the absorption maxima of the bound xanthophylls [19] (see legend to Table 3). Thus, it is possible, by applying a spectral deconvolution analysis, using spectral forms of Chl and carotenoids in protein environment [20] to deduce the protein environment in which a carotenoid is bound. The complete data set for spectral deconvolution is given [see Additional file 8], while relevant results are summarized in Tab. 3.

Since, in LHCII monomers from *lut2.1*, lutein is completely substituted by violaxanthin, we asked if this xanthophyll occupies the same sites L1 and L2 occupied by lutein in the WT. We used for this analysis IEF-purified LHCII proteins, in which the external V1 site is empty [19]. The results are summarized in Table 3. The low amplitude Viola spectral form at site L2 (492 nm) [18] is maintained in *lut2.1 *with a 4-fold higher amplitude, meaning that this site is now completely occupied by violaxanthin. A new violaxanthin spectral form, with a similar amplitude and an unusually high red-shift (505 nm) appears at site L1. Neoxanthin spectral forms and energy transfer are instead unaltered in *lut2.1 *with respect to WT. Both violaxanthin spectral forms in *lut2.1 *show high efficiency of energy transfer (80–90%) to Chl *a*. Since energy transfer is strongly influenced by the pigments' mutual distance and orientation, these data strongly suggest that the two violaxanthins occupy, in *lut2.1*, the L1 and L2 sites. The unusually high red-shift and energy transfer efficiency of Viola at site L1 is probably due by the "unnatural" binding of this pigment at this site, normally occupied by lutein.

### Unaltered thermal stability of purified Lhcb proteins binding violaxanthin

In order to identify a possible effect of the altered pigment composition on the stability of Lhc proteins, we measured the heat denaturation dependence of the major CD signal at 492 nm [21,22] [see Additional file 7]. In band 2 from *lut2.1 *and WT, two inflection points showing essentially the same values were found, suggesting that both LHCII and minor Lhcb complexes had, on the average, the same stability to heat denaturation, irrespective of whether they bound violaxanthin or lutein. In order to distinguish between the contributions of individual Lhc gene products to the above determination, the band 2 from WT and *lut2.1 *was fractionated by preparative IEF and the fractions analyzed for polypeptide composition [see Additional file 9]. Fractions containing the same Lhcb apoproteins, as determined by SDS-PAGE, were analyzed for their stability to heat denaturation and their pigment composition [see Additional file 3]. It clearly appeared that not only LHCII, but also other Lhcb proteins folded correctly and showed unaltered stability when violaxanthin was substituted for lutein. The Chl *a/b *ratio was significantly lower in LHCII isoforms from *lut2.1 *with respect to WT, while IEF bands with less acidic pI, enriched in minor Lhc proteins, were less affected in their Chl *a/b *ratio.

### Photoprotection and carotenoid triplet formation in lutein vs violaxanthin-binding Lhc proteins

**Figure 2:**
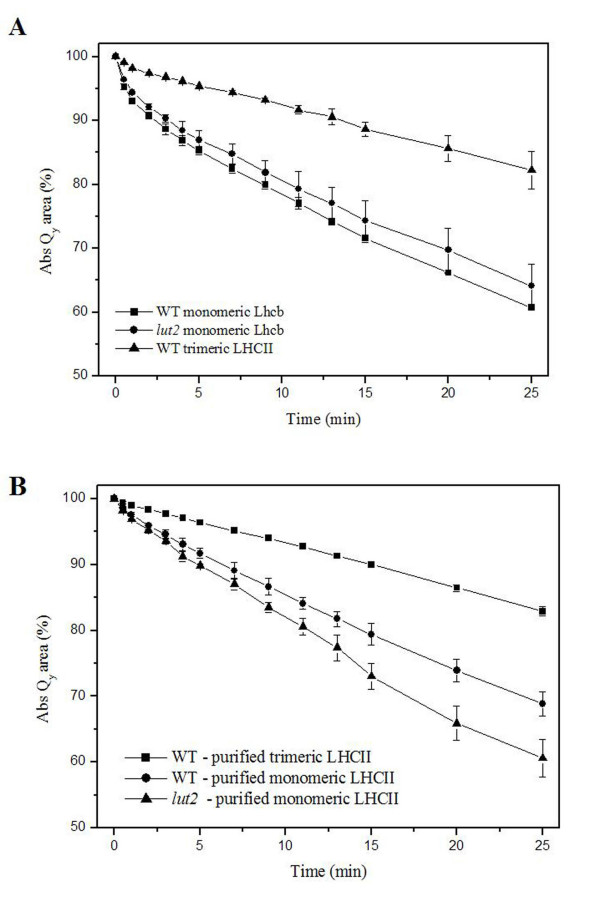
**Photobleaching behaviour of isolated Lhcb**. (A) Monomeric Lhcb isolated from solubilized thylakoids of WT and *lut2.1*, and trimeric LHCII from WT were analyzed by following the Q_y_-transition absorbance decay during strong illumination. (B) Sucrose bands 2 and 3 from WT and *lut2.1 *were fractionated by flat bed IEF in order to purify LHCII subunits in their monomeric and trimeric form. Kinetics of Q_y_-transition absorbance decay were measured on isolated complexes as described in Experimental Procedures. Chlorophyll concentrations of Lhcb were set to 8 μg/ml. Samples were cooled to 10°C during measurements.

Strong illumination of chlorophyll-proteins in the presence of oxygen leads to ^3^Chl* formation, which reacts with molecular oxygen forming ^1^O_2_*. Singlet oxygen causes bleaching of Chl with kinetics inversely dependent on the efficiency of chlorophyll triplet quenching by bound xanthophylls. The photobleaching behavior of pigment-proteins from sucrose gradient bands (Figure 1A) was determined as previously described [9]. The results are shown in Figure 2A. The highest resistance was found in WT band 3, containing trimeric LHCII, while band 2, containing mostly minor Lhcbs, was more prone to photobleaching in agreement with previous findings [23]. In the case of band 2 from *lut2.1*, the resistance to photobleaching was, surprisingly, only slightly higher than in the case of WT, although the LHCII content was much higher (the LHCII/minor antennae ratio was 2.5 in band 2 from WT and 3.7 in *lut2.1*, see Figure 1B). This suggests that either the presence of violaxanthin, rather than lutein, within these proteins, or the monomerization of LHCII, caused a decreased resistance to photobleaching. To clarify this point, we analyzed the photobleaching behavior of monomeric LHCII from WT and *lut2.1 *purified by IEF (Figure 2B). The *lut2.1 *complex was clearly more sensitive to photobleaching than that from WT. An increase in resistance to photobleaching was detected in trimeric LHCII from WT with respect to the monomeric form, thus indicating that trimerization *per se *contributes to photoprotection. Into LHCII, site L1 was shown to be essential for ^3^Chl* quenching and consequently for protection from photobleaching in the presence of oxygen, while site L2 had little relevance in this respect [9]; therefore, we conclude that the reduced resistance to photobleaching is due not only to the monomerization of LHCII subunits, but also to the substitution of lutein in site L1 by violaxanthin.

**Figure 3:**
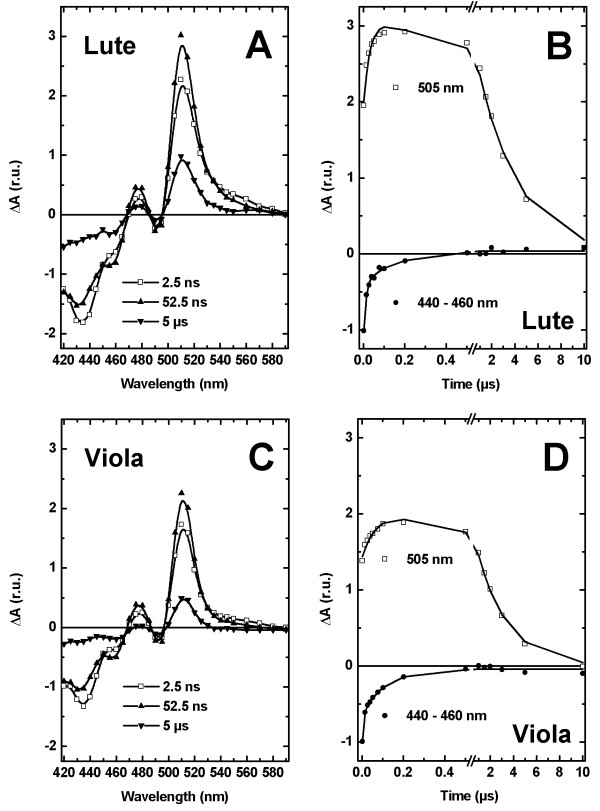
**Flash-induced absorbance changes due to carotenoid triplet formation in LHCII recombinant proteins **reconstituted with lutein (panels A and B) or violaxanthin (panels C and D). Panels A and C show the complete difference spectra recorded at different time points (2.5 ns, 52.5 ns and 5 μs). Panels B and D show absorbance changes at 505 nm (^3^Car*) and 440–460 nm (*Chl). Data have been normalized on the amount of excited chlorophyll measured at 440 – 460 nm, and fitted to a biphasic model (solid symbols in panels B and D).

In order to further substantiate this conclusion, we performed direct measurements of the kinetics of carotenoid triplet formation and triplet chlorophyll quenching by time-resolved spectroscopy of lutein- *vs*. violaxanthin-containing monomeric Lhcb1 proteins. Time-resolved absorbance changes were recorded, subsequently to chlorophyll excitation at 650 nm. Consistent with previous results [9] recombinant proteins binding violaxanthin showed faster photobleaching than those binding lutein (not shown). The data shown in Figure 3 refer to *in vitro *reconstituted, recombinant proteins. ^3^Car* formation and decay can be followed as the changes in absorbance at 505 nm, while ^1^Chl* gives a negative signal at 440–460 nm (panels B and D, see Experimental Procedures for a detailed discussion of the spectral deconvolution procedure). Spectra measured on lutein- and violaxanthin-containing Lhcb1 gave similar half-times for ^3^Car* decay (2–2.5 μs) but evidenced a rise-time for violaxanthin triplet (~50 ns) slower than for lutein (~20 ns). Analysis of purified monomeric LHCII proteins purified from WT and *lut2.1 *membranes by IEF yielded similar results (data not shown).

### The effect of high light growth conditions

**Figure 4:**
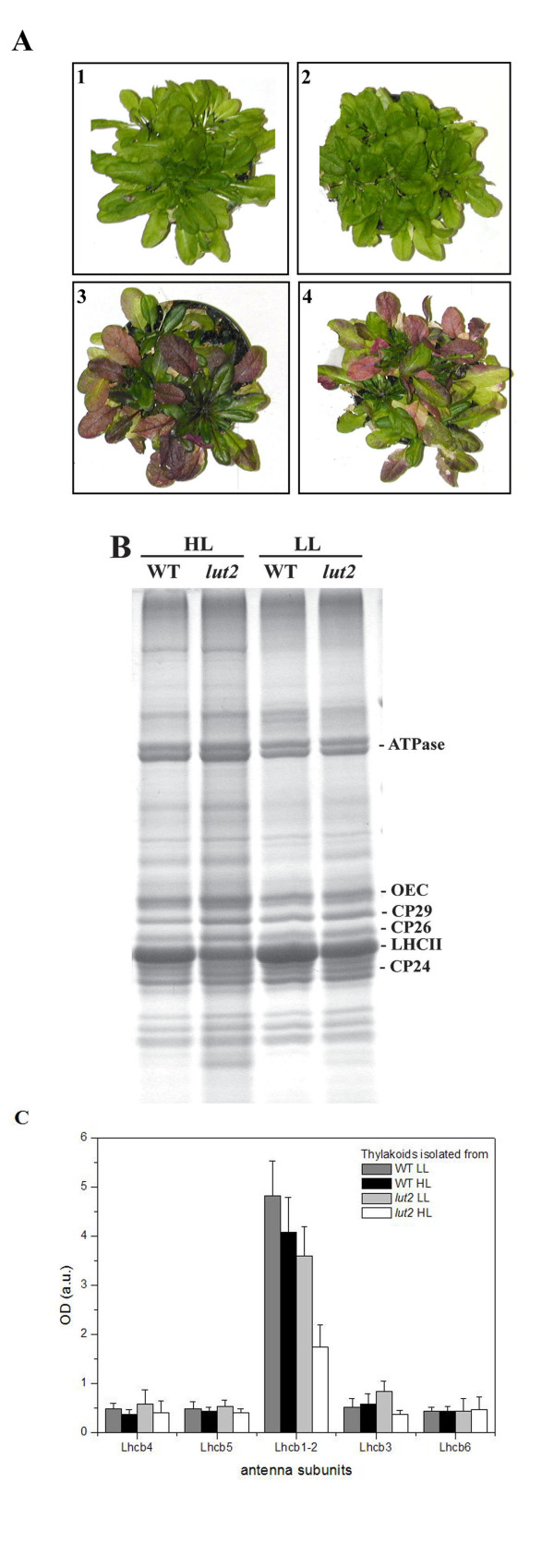
**Phenotypes of WT and *lut2.1 *grown in normal and high light conditions**. (A) Three-weeks-old WT (Fig. 1,3) and *lut2.1 *(Fig. 2,4) plants were grown for 3 additional weeks in normal light conditions (21°C, 120 μmol m^-2 ^s^-1 ^- LL) (Fig. 1,2) or in high light conditions (21°C, 1400 μmol m^-2 ^s^-1 ^- HL) (Fig. 3,4). (B) Tris-Tricine SDS-PAGE analyses of thylakoid from LL or HL plants. Main protein components are indicated. (C) Relative level of thylakoid antenna proteins evaluated by densitometry of bands identified by immunoblotting.

The biochemical data suggest a deficit in the efficiency of photoprotection at the level of Lhcb proteins, particularly LHCII, in the *lut2.1 *mutant, caused by the substitution of lutein with violaxanthin in site L1. It can thus be expected that growth at high light intensity may reveal additional features of the *lut2.1 *phenotype. WT and *lut2.1 *plants were grown for 3 weeks in control conditions (120 μmol m^-2 ^s^-1^) at 21°C and then either exposed to high light (1400 μmol m^-2 ^s^-1^) or grown at the same light intensity for three additional weeks (Figure 4A). After treatment, leaves were analyzed for pigment composition [see Additional file 4] and thylakoid protein composition (Figure 4B–C). Growth in high light produced damages consisting into reddening and bleaching of older leaves. The damages were more pronounced in mutant plants.

Thylakoid membranes were isolated from low- and high-light grown plants and analyzed by SDS-PAGE (Figure 4B–C). The relative abundance of thylakoid proteins was evaluated by densitometry of Coomassie-stained gels upon identification of individual selected bands by immunoblotting with specific antibodies (not shown). Both WT and *lut2.1 *thylakoids showed a decrease in the LHCII/PSII ratio in high light, as evaluated by the level of the 33 kDa oxygen evolving complex 1 polypeptide (Figure 4B). WT plants decreased their content in Lhcb1+2 polypeptides upon growth in high light by 15% with respect to control plants while other Lhcb proteins were marginally affected. *lut2.1 *plants showed a similar effect, but the amplitude of the decrease in LHCII was much higher, suggesting that mutant plants over-react to increasing light by degrading their major antenna complex and thus avoiding photoinhibition (Figure 4C).

In agreement with previous results [12] the Chl *a/b *ratio increased in WT and *lut2.1 *with respect to control conditions, the amplitude of the change being higher in the mutant. *lut2.1 *had increased Chl *a/b *ratios even in control conditions. Growth in high light decreased the Chl/Car ratio in WT and *lut2.1*. WT plants did not contain any A+Z in low light, and low levels in high light conditions. *lut2.1 *plants contained low, but detectable levels of A+Z in low light conditions [14], and their increase in high light was 8 times higher than in WT plants. Although the increase in A+Z was the highest, all carotenoid species increased their relative amount with respect to Chls. This effect was stronger in *lut2.1 *with respect to WT plants [see Additional file 4].

### Photooxidation at low temperature

Our results strongly suggest that *lut2.1 *plants are affected in their capacity to prevent photooxidation of their antenna system, due to the lower efficiency of violaxanthin, with respect to lutein, in quenching ^3^Chl*. Growth in low temperature conditions should enhance the amplitude of photodamage [24]. We have thus evaluated the effect of growing plants at 4°C at either low (20 μmol m^-2 ^s^-1^) or high light conditions (800 μmol m^-2 ^s^-1^). The experiment was performed on WT, *lut2.1*, *npq1 *(previously shown to have a decreased resistance to oxidative stress under light stress conditions [6]) and the double mutant *npq1lut2.1*. While WT and *lut2.1 *are able to increase A+Z content at the expense of Viola upon light treatment, *npq1 *and *npq1lut2.1 *plants cannot [see Additional file 1].

**Figure 5:**
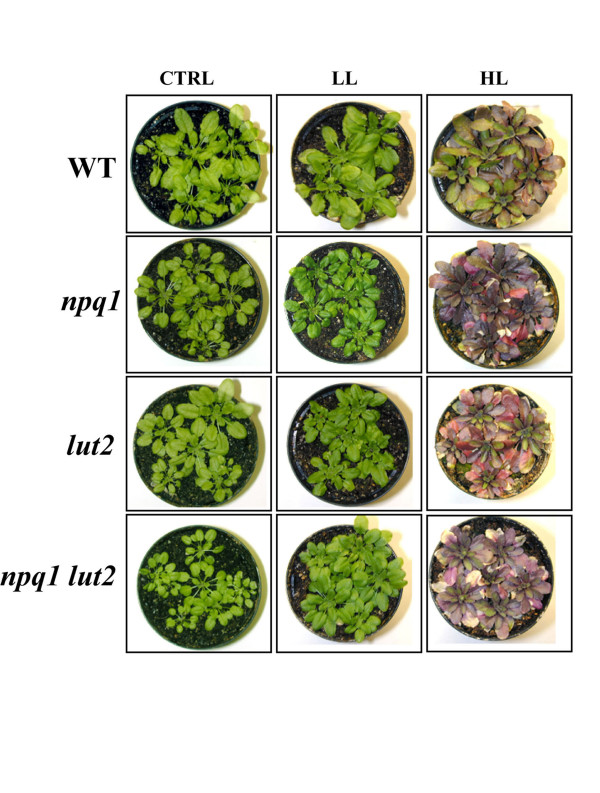
**Phenotypes of WT and different mutants grown in different light conditions at 4°C**. Plants were growth for 3 weeks in control conditions (21°C, 120 μmol m^-2 ^s^-1^, (CTRL) and then transferred for 3 additional weeks at 4°C at either 20 μmol m^-2 ^s^-1 ^(LL) or 800 μmol m^-2 ^s^-1 ^(HL).

Plants were grown at 120 μmol m^-2 ^s^-1^, 21°C for three weeks (t_o_) and then transferred at 4°C at either low light or high light for three additional weeks. In low light, none of the genotypes showed an evident stress effect, while in high light, plants were affected to different extents (Figure 5): in WT, older leaves showed photobleaching accompanied by accumulation of anthocyanin, an indicator of stress in Arabidopsis [25,26]. These symptoms were much stronger in *npq1 *and *lut2.1 *mutants, extending to the younger leaves, while many of the older leaves were almost completely bleached. Consistently with previous reports [27], the *npq1lut2.1 *genotype was more light-sensitive than either *npq1 *or *lut2.1*, suggesting that the lack of zeaxanthin exacerbates the photodamage induced by the lack of lutein. More quantitative analyses were performed on detached leaves, choosing leaves that remained green over the entire period of the experiment [see Additional file 5].

**Figure 6:**
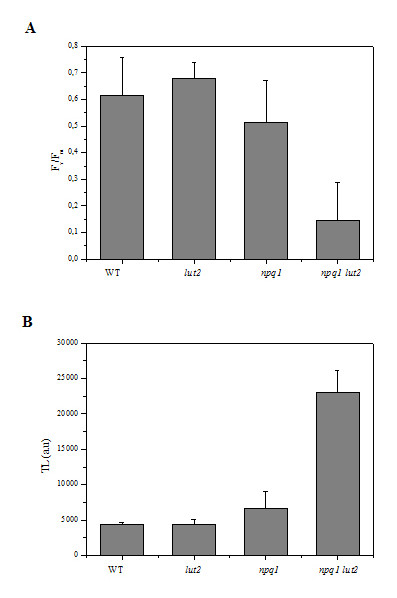
**Analyses of the sensitivity to photoxidative stress on WT and mutant genotypes**. Whole plants (WT and different mutants) were exposed for 30 hours to a high photon flux density (1100 μmol m^-2 ^s^-1^) at low temperature (10°C). (A) Measurements of the chlorophyll fluorescence ratio F_v_/F_m_, an index of photoinhibition of PSII. (B) Analyses of heat-induced luminescence emission of WT and mutant leaves; the 135°C emission band was used as an index of lipid peroxidation and oxidative stress.

Plants of WT and mutants, grown in standard conditions (120 μmol m^-2 ^s^-1^) were treated for 30 hours at high light and low temperature (1100 μmol m^-2 ^s^-1^, 8 hours light photoperiod, 8°C). Following stress, the level of photoinhibition was assayed by chlorophyll fluorometry (F_v_/F_m_) (Figure 6A), while lipid peroxidation was quantified by measuring leaf chemiluminescence [28,29] (Figure 6B). Our results clearly show that the highest levels of lipid peroxidation and photoinhibition were obtained in the *npq1lut2.1 *genotype, in accordance with evidences obtained on *C. reinhardtii lor1npq1 *double mutant [30]; *npq1 *had intermediate levels and *lut2.1 *did not show a significant difference from WT. Similar results were obtained in a shorter experiment in which detached leaves, floating in water at 10°C, were treated at high light (1100 μmol m^-2 ^s^-1^) for 20 h (data not shown).

## Discussion

**Figure 7:**
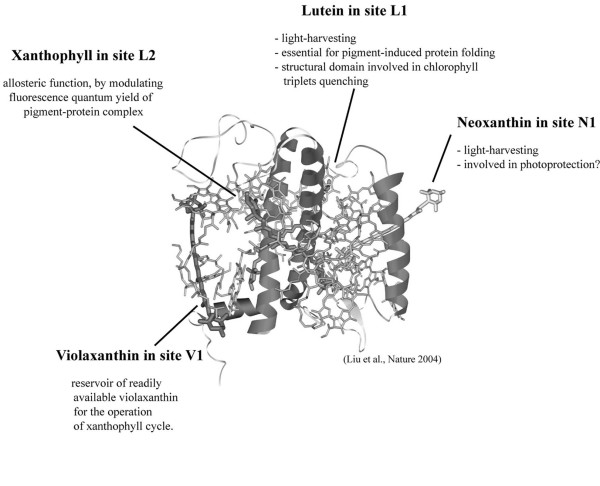
**Proposed role of xanthophylls in higher plant antennae**. LHCII structure is taken from [17].

The conservation of plant xanthophyll composition strongly suggests that each xanthophyll species has a specific function. Lutein is the major xanthophyll species in plants, accounting for approx. 60% of total xanthophylls and 40% of total carotenoids in leaves. In LHCII complexes, it binds to site L1, whose occupancy is essential for protein folding and chlorophyll triplet quenching, and, promisquously with other xanthophylls, site L2, essential for photoprotection by violaxanthin/zeaxanthin exchange [9] (Figure 7). Still, it has been reported that lutein is not essential for photosynthesis [14]. Additional studies have shown alterations, in the *lut2 *mutant, in NPQ, LHCII antenna size and trimerization, and an increased accumulation of A+Z [31] while and recent publication showed decreased growth rate in a large range of light conditions. We have confirmed and extended some of these observations (see Additional files). It is worth noting that our *lut2.1 *mutant was isolated in Wassilewskija genetic background, while previous described lutein-less mutant [12,14] are in the Columbia ec. It seems proper to ask if differences between our and previous results are related to the different genetic background. We have addressed this question by confirming in Wassilewskija ec. results previouly obtained in Columbia ec. We concluded that the level of sensitivity to stress and other photosynthetic parameters were the same in boh ecotypes. Furthermore, we obtained several confirmatory results using *lut2.1 *mutant, which closely match those previouly obtained in the Columbia ec. [12]. We conclude that the two mutants are, in every respect, comparable. Finally, in a later stage of the study, we succeeded in isolating an equivalent mutant from the Columbia background [32] which had the same properties as those described here for *lut2.1*.

A complete disruption of the LHCII trimeric organization was observed in the *lut2.1 *mutant even upon solubilization of thylakoids with the mild detergent α-DM, which is very effective in retaining trimers in WT. Protein gel analyses of purified LHCI and LHCII monomers show that they have unaltered protein composition, and HPLC analyses show that only violaxanthin and neoxanthin are bound to LHCII complexes. Previous work with recombinant proteins has shown that lutein, violaxanthin and zeaxanthin can bind to sites L1 and L2 of Lhc proteins [18,33] while the site for neoxanthin binding is site N1. This was recently confirmed by X-ray crystallography [17]. We found a novel, red-shifted form of violaxanthin in LHCII from *lut2.1*, consistent with the red-shift observed for lutein in site L1 of WT LHCII [18]. This strongly suggests that, in *lut2.1*, violaxanthin replaces lutein in site L1.

LHCII from *lut2.1 *contains more than one neoxanthin molecule per polypeptide suggesting that this xanthophyll can compete with violaxanthin in either sites L1 or L2. Since reconstitution with neoxanthin only was unable to yield a pigment-protein complex in all Lhc proteins, and occupancy of site L1 was shown to be needed for refolding [9,34,35], we conclude that, in LHCII, neoxanthin can compete with violaxanthin for site L2 in the absence of lutein. This is consistent with previous results [36] obtained *in vitro *using low stringency reconstitution of recombinant proteins and with the binding of neoxanthin to L2 site in the homologous proteins CP29 and CP26.

LHCII monomerization appears to be due to the lack of lutein in sites L1 and/or L2 *per se*, as indicated by *in vitro *reconstitution and trimerization of the *Lhcb1 *apoprotein. We demonstrated, for the first time, that trimers can only be formed if lutein is present in the reconstitution mixture. Any substitution of lutein with other xanthophyll species leads to monomerization, as shown also for the *npq2lut2.1 *mutant [37]. Violaxanthin-binding LHCII monomers have the same stability to heat denaturation as lutein-binding monomers, implying that binding of violaxanthin impairs trimerization but not protein stability. Violaxanthin-containing LHCII from *lut2.1 *is equally efficient in light harvesting, since the small decrease in functional antenna size in *lut2.1 *is quantitatively consistent with the lower LHCII content determined by biochemical methods.

*lut2.1 *plants were unable to perform state I – state II transitions. We first considered the possibility that the preferential absorption of far-red light by the PSI-LHCI complex, which is at the basis of state I – state II transitions, could be somehow affected by the absence of lutein. This possibility was ruled out by the observation that, consistent with previous results in Chlamydomonas PSI [38], the PSI-LHCI complex in sucrose gradient ultracentrifugation was very stable and conserved the red-shifted absorption tail typical of WT complex (data not shown). The loss of state transitions could either be due to the specific loss of a particular LHCII subpopulation able to migrate from grana to stroma membranes [39] or an impaired capacity for LHCII phosphorylation in the mutant. The first hypothesis is ruled out by the results of non-denaturing IEF analyses showing the presence of the same isoforms in WT and *lut2.1*. We suggest that LHCII phosphorylation is somehow affected in *lut2.1*. Phosphorylation experiments are in progress in order to verify this hypothesis. Alternatively, the modified conformation of violaxanthin-binding LHCII might affect the conformational changes [40], which are involved in detachment from PSII and docking to PSI [41].

Recent reports have suggested the hypothesis that trimer to monomer transition is the basis of NPQ [42]. Since *lut2.1 *LHCII is completely monomeric, but still shows residual NPQ, the data contradict this hypothesis. Recent work has shown that NPQ is first elicited in the PSII core complex and is then propagated to the antenna system [43]. Quenching in isolated LHCII has been proposed to be catalyzed by interactions between chlorophyll molecules bound to binding sites A1 and/or A2 with the lutein in site L1, elicited by a conformational change [44]. Thus, the substitution of lutein by violaxanthin in site L1 may limit the efficiency of the process.

WT and *lut2.1 *plants grow similarly in moderate light conditions according to an early report [14]. In high light, stress effects such as anthocyanin accumulation and bleaching of older leaves were more pronounced in *lut2.1 *plants. Pigment composition clearly showed that the increase in carotenoids on a Chl basis was stronger in *lut2.1 vs*. WT. These changes are commonly observed upon exposure to light stress [45] and correlate with an increased resistance to excess light [46]. Further reactions to excess light consist into a decrease in PSII antenna size [47] by specific proteolysis of the LHCII complex [48]. Determination of Lhc protein content in thylakoids of WT and *lut2.1 *plants showed that the amount of LHCII was lower in *lut2.1 *grown in low light and that further decrease upon exposure to high light was higher in the mutant with respect to WT plants. This is possibly due to the accumulation of zeaxanthin in *Lhc *complexes, favoring degradation of the major LHCII complex [37]. Thus, the *lut2.1 *mutant is more sensitive to light than WT and overreacts to an increase in light intensity through the enhanced operation of known mechanisms of photoprotection [49]. The over-operation of these mechanisms likely compensates for the primary lesion brought about by the lack of lutein.

What is the primary effect of the *lut2.1 *mutation? Previous work with recombinant proteins obtained by reconstituting *in vitro Lhc *apoproteins with different xanthophylls, has shown that LHCII binding violaxanthin in sites L1 and L2 undergoes a more rapid photobleaching when illuminated in the presence of oxygen with respect to the LHCII binding lutein [9,18]. Photobleaching is the effect of the ^1^O_2_* produced by the reaction of ^3^Chl* with molecular oxygen, which is a triplet in its ground state. *In vivo*, ^3^Chl* is produced by intersystem crossing from ^1^Chl* and is efficiently quenched by carotenoids, leading to heat dissipation of triplet energy [5]. LHCII purified from *lut2.1 *is more prone to photobleaching than the complex from WT. Since the fluorescence quantum yield, and thus the ^1^Chl* concentration on LHCII binding Viola + Zea is essentially the same, we conclude that violaxanthin is less efficient than lutein in quenching ^3^Chl*, thus resulting in increased ^1^O_2_* formation and photobleaching.

The first excited triplet state of carotenoids lies below the energy level of chlorophyll triplet and singlet oxygen. Therefore, the generation of ^3^Car* can quench ^3^Chl* and scaveng ^1^O_2_*. ^3^Car* decay into the ground state without emission of radiation, and thus act as safe repositories of excess energy. The increased photobleaching of *lut2.1 *suggests that, for the same amount of ^3^Chl*, the relative concentration of ^1^O_2_* is increased. This could be due to a decreased formation of ^3^Car*. As ^3^Car* decay is very fast (~2 μs) we addressed this question by flash-induced, time-resolved spectroscopy. Violaxanthin triplets exhibit an extinction coefficient (from 3·10^5 ^to 6·10^5 ^at 490 nm) larger than lutein (about 2·10^5 ^at 500 nm) [50]. Despite this difference, after normalization for the *Chl signal, we observed that absorbance changes due to lutein triplet formation were 1.5-fold larger than for violaxanthin, and the kinetics for triplet formation were faster for lutein. This proves that lutein bound to LHCII proteins is more efficient as a ^3^Chl* quencher than violaxanthin.

The binding site responsible for the increased ^1^O_2_* production is, likely, site L1, since the non-occupancy of site L2 did not significantly affect photobleaching in recombinant LHCII [9,18]. It is possible that the different conformation of LHCII protein binding violaxanthin modify the Chl-to-xanthophyll distance, which is crucial for triplet quenching [51]. This effect is independent from the stability of LHCII protein folding as assessed by thermal denaturation, but is dependent on the aggregation size of the complex, implying that trimerization increases the "special" Chl to carotenoid interaction responsible for optimal photoprotection. This hypothesis is supported by the report that monomerization of LHCII yields into the loss of a specific red-shifted (510 nm) spectral form of lutein [19].

Thus, the primary lesion in *lut2.1 *mutation is the enhanced production of singlet oxygen in the major PSII antenna complex, partially compensated by increased zeaxanthin production. In order to verify this hypothesis, we have studied the behavior, under enhanced stress conditions (high light + low temperature), of the *lut2.1 *mutant compared to the double mutant *npq1lut2.1*, which cannot synthesize zeaxanthin upon exposure to excess light. Zeaxanthin free in the membrane, upon release of violaxanthin from LHCII and de-epoxidation by VDE, has been suggested to protect from photooxidation, by a ROS scavenging effect [6,30] that supplements the action of vitamin E [52]. A previous paper [27] reported phenotipic evidences of a higher sensitivity of double mutant *npq1lut2 *to photoxidative stress. We performed a detailed description of this genotype, showing both its behaviou during short high-light treatment, and acclimation to long-term stress. The *npq1lut2.1 *plants underwent stronger photoinhibition, anthocyanin biosynthesis and lipid oxidation not only with respect to WT but also with respect to both *npq1 *and *lut2.1 *single mutants. Our results Thus point to a strong photosensitive phenotype in higher plant mutant due to to lack of both lutein and zeaxanthin; such data are in agreement with those obtained in *C. reinhardtii npq1lor1 *mutant [30]. In contrast, a recent publication [13] failed to evidence a synergistic effect of lutein and zeaxanthin in promoting growth and preventing stress; these results could be ascribed to the different setting of stress conditions used with respect to our work. Our data, in agreement with [27] and [30], let to conclude that zeaxanthin is effective in photoprotection of plants lacking lutein. This is due to the multiple effects of zeaxanthin in photoprotection, including ROS scavenging [6,30,53] and direct quenching of Chl fluorescence by binding to the L2 allosteric site of Lhc proteins [54].

## Conclusion

The conservation of carotenoid composition across the plant kingdom implies a specific function for each xanthophyll species. Lutein has the specific property of quenching harmful ^3^Chl* by binding at site L1 of the major LHCII complex and of other Lhc proteins of plants, thus preventing ROS formation. Substitution of lutein by violaxanthin decreases the efficiency of ^3^Chl* quenching and causes higher ROS yield. The phenotype of *lut2.1 *mutant in low light is weak only because rescuing mechanisms of photoprotection, namely zeaxanthin synthesis, compensate for the ROS production, as also supported from the lower antenna size observed in low light. Thus, the light sensitive phenotype becomes evident in conditions enhancing photooxidative stress or when the additional mechanisms are eliminated by the *npq1 *mutation. Excess light and low temperature are commonly experienced by plants during their lifecycle. Therefore, performing photosynthesis without lutein is like driving without a seat belt. At low speed, one can get away with it as additional protective mechanisms (airbags = zeaxanthin) partially compensate for the damage. But, in stress conditions, the additional mechanisms fail and the damage becames evident.

The specificity of site L1 for lutein in all Lhc proteins [10] probably derives from the co-evolution of the carotenoid biosynthesis pathway and Lhc proteins with multiple binding sites dedicated to different functions. Lutein is the only xanthophyll containing one beta and one epsilon ring. Beta and epsilon carotenoid cyclases have diverged early during plant evolution, and are both found in all higher plant taxa, as well as in green algae like Scenedesmus or Chlamydomonas [55]. In most plants, lutein is not undergoing epoxidation-deepoxidation reactions in response to environmental conditions, while the beta-beta xanthophylls do. Since site L1 is dedicated to the essential function of Chl triplet quenching, it may be desirable, for the plant, that the chromophore catalyzing this function is not involved in rapid concentration changes in response to environmental conditions as happens for the beta-beta xanthophylls involved in the xanthophyll cycle [56].

## Methods

### Screening procedure

We analyzed 20000 independent T-DNA insertion lines of *Arabidopsis thaliana *(accession Wassilewskija-2) available from the Institut National de la Recherche Agronomique (INRA, Versailles, France). Four hundred seed pools, each representing one row or one column of the grid (100 lines), were prepared and then combined into 80 superpools, each representing 500 lines.

The presence of T-DNA insertion in the lycopene ε-cyclase gene (*lyec*) was assessed by PCR amplification on DNA from each of the superpools, followed by a nested PCR. The first (10 cycles: 94° 2'; 65°-1°/cycle 30"; 72° 2'. 35 cycles: 94° 15"; 55° 30"; 72° 1') was performed by using the *lyec *specific primers 5'-AGTTAGTCGACGTTTGCTCCATG-3' and 5'-CAATGGTAATAGGCTTGTCATC-3' and the T-DNA specific primers 5'-CTACAAATTGCCTTTTCTTATCGA-3' and 5'-CTGATACCAGACGTTGCCCGCATAA-3' The nested PCR (35 cycles: 94° 45"; 56° 45"; 72° 1') was performed by using the *lyec *specific primers 5'-GAGGAGGTAAAGTATGGTTCCAC-3' and 5'-CTCTCTCCAAACATGCTCAATAC-3' and the T-DNA specific primers 5'-CATGTACATCAAGCTTATCGATAC-3' and 5'-TACGAATATCTGCATCGGCGAAC-3'. One mutant line, containing a T-DNA insertion in the sixth exon of the gene, was identified in the superpools and then in the pools, and kindly provided by INRA. To identify homozygous lines, PCR analyses was performed using primers 5'-AAGCTTCTTCCGTACTTTC-3' and 5'-CAATCGTAAACAATATAAGCG-3', flanking the site of insertion, and the T-DNA specific primer 5'-CATGTACATCAAGCTTATCGATAC-3'. This mutant will be indicated as *lut2.1 *in order to avoid confusion with the original *lut2 *mutant [14] solely to indicate that it is made in Wassilewskija ecotype. After completion of this work we have obtained the same mutation in Columbia ecotype and verified that it showed the same behaviour as the *lut2.1 *with respect to sensitivity to light stress.

### Plant material

WT plants of *Arabidopsis thaliana *ecotype Wassilewskija and mutants *npq1 *[57], were obtained from the Arabidopsis Stock center. Genotype *npq1 lut2.1 *was obtained by crossing single mutant plants. Plants were grown for three weeks in controlled conditions (~120 μmol m^-2 ^s^-1^, 21°C, 8 h light/16 h dark). For long term treatment, 3 weeks old seedlings were exposed (a) to light conditions of 120 or 1400 μmol m^-2 ^s^-1 ^for 3 weeks at 21°C, and (b) to light conditions of 20 or 800 μmol m^-2 ^s^-1 ^for 3 weeks at 4°C. Short-term high light treatment was performed for 20 minutes at 1200 μmol m^-2 ^s^-1^.

### Chlorophyll fluorescence and photosynthetic oxygen evolution in vivo

Chlorophyll fluorescence from intact leaves or from leaf discs was measured with a PAM-2000 fluorimeter (Walz), as previously described [37]. The maximal quantum yield of PSII photochemistry was measured in dark-adapted leaves from the maximal fluorescence level (F_m_) and the initial level (F_o_): (F_m_-F_o_)/F_m _= F_v_/F_m_. Variable fluorescence was induced in leaf discs, infiltrated with DCMU 2.5 10^-5 ^M, with a red light of 8 μmol m^-2 ^s^-1 ^produced by a light emitting diode. The half-time of the fluorescence rise was taken as a measure of the functional antenna size of PSII [15]. Photosynthetic O_2 _evolution was measured with the photoacoustic method, as described by Havaux *et al*., [37] (for a review, see [58]). The Emerson enhancement (E) of O_2 _evolution was determined in state I or in state II by adding a continuous far-red light (>715 nm, 34 W m^-2^) to the modulated blue-green light (obtained with a BG38 Schott filter; photon flux density 24 μmol m^-2 ^s^-1^). E (%) = [(Φ (+FR) - Φ (-FR))/Φ (-FR)] × 100, where Φ (+FR) is the amplitude of oxygen evolution signal in the presence of the far-red light and Φ (-FR) is the signal measured with the modulated exciting light only. State II was reached by illuminating leaves with blue-green light for 10 min, and state I was obtained after 10-min illumination with far-red light.

Non-photochemical quenching of chlorophyll fluorescence was measured with a PAM 101–103 fluorimeter (Walz). NPQ was calculated according to the following equation [59]: NPQ = (F_m_-F'_m_)/F'_m_, where F_m _is the maximum Chl fluorescence from dark-adapted leaves and F'_m _the maximum Chl fluorescence under actinic light exposition.

### Thylakoid isolation and sample preparation

Unstacked thylakoid membranes were isolated from dark-adapted leaves as previously described [39].

Membranes corresponding to 500 μg of chlorophylls were washed with 5 mM EDTA and then solubilized in 1 ml with 0.6% α-DM, 10 mM HEPES pH 7.5. Solubilized samples were then fractionated by ultracentrifugation in a 0.1–1 M sucrose gradient containing 0.06% α-DM, 10 mM HEPES pH 7.5 (22 h at 280,000 × g, 4°C).

Monomeric Lhcb proteins were further fractionated by flat-bed isoelectric focusing at 4°C as previously described [60].

### Pigment analyses

The pigments were extracted either from whole leaves, thylakoid membranes and isolated antenna complexes with 80% acetone, then separated and quantified by HPLC [61] and by fitting of the spectrum of the acetone extract with the spectra of individual pigments [21].

### Gel electrophoresis

SDS-PAGE analyses was performed with the Tris-Tricine buffer system as previously described [62]. Gel images were acquired using a Bio-Rad GS710 scanner. The picture was then analysed with GEL-PRO ANALYZER software (Media Cybernetics Inc., MD, USA) that quantifies the staining of the bands as IOD (optical density integrated on the area of the band).

### In vitro reconstitution of LHCII pigments complexes and trimerization assay

The construct over-expressing Lhcb1 was obtained as described [63], except for a sequence coding for a His_6 _tail inserted at the 3' end before stop codon. *In vitro *reconstitution of LHCII with altered xanthophyll composition was performed as previously described [18]. Purification of reconstituted holocomplexes was performed by a Ni^2+ ^chelating column (Pharmacia Source 15S) as described [9]. Reconstitutions were accomplished with a mix of xanthophylls as follow: LHCII Viola, 100% violaxanthin; LHCII Lute, 100% lutein; the Chl *a/b *ration in the mixture was 2.3. In order to perform *in vitro *trimerization of reconstituted complexes, we followed a method previously described [64] with some modifications. Ni^2+ ^column and bound reconstituted LHCII were washed with trimerization buffer: 0.1 mg/ml PG, 0.06% β-DM, 0.2 M NaCl, 20 mM phosphate buffer pH 7.5, 10 mM imidazole. Pigment-protein complexes were collected by washing column with eluting buffer: 0.5 M imidazole, 20 mM phosphate buffer pH 7.5, 0.2 M NaCl, 0.06% β-DM; LHCII trimers were separated from monomers by sucrose gradient ultracentrifugation [19].

### Spectroscopy

Steady state spectra were obtained using samples in 10 mM HEPES pH 7.5, 0.06% α-DM, 0.2 M sucrose. Absorption measurements were performed using a SLM-Aminco DW-2000 spectrophotometer at RT.

Time-resolved spectroscopy: absorbance changes were monitored with a home-built pump and probe laser spectrophotometer, basically described in [65], and modified as follows. The wavelength of monochromatic 10-ns light pulses used for the detection of absorbance changes is tuned from 410 nm to 590 nm by an optical parametric oscillator pumped by the third harmonic of a Nd:YAG laser (Surelite, Continuum). The 10-ns excitation flash is provided by a home-built broadband dye laser cell filled with DCM in methanol (650 nm) and pumped by the second harmonic of a Nd:YAG laser (Minilite, Continuum). The delay between the pump and probe pulses is adjusted with a 100 MHz National Instruments PCI-6552 digital waveform generator. Detection beam is split in two ahead of measure and reference 10 × 10 mm cuvettes. Transmitted light is filtered through a combination of 6 mm-thick BG39 colored Schott filter and CVI low-pass dielectric filter (600 nm). Light intensity is measured with large area silicon photodiodes fitted to AC-coupled preamplifiers. The difference between measure and reference analog signals is achieved by a Tektronix differential amplifier (gain 10). Acquisition of difference and reference signals is performed *via *a 16-bit National Instrument digitizer PCI-6052E. Optical density of the sample was adjusted to 1 in the Qy band of chlorophylls. The flash-induced signal exhibits negative peaks around 430 nm, characteristic of the bleaching of the carotenoid S_2_←S_0 _transitions, and a strong positive peak around 510 nm attributed to the carotenoid T_2_←T_1 _transition. The spectra are thus denoted triplet-minus-singlet difference absorption spectra. The extent of the carotenoid absorbance change at 5 μs is approximately the same at 440 nm and 460 nm. This led to the choice of these two wavelengths as isosbestic points of the carotenoid TmS spectrum. At 2.5 ns, a contribution of excited chlorophyll (Chl*) could be detected within the time-resolution of the instrument, contributing negatively at 440 nm and positively at 460 nm. Consequently, the kinetics of pure Chl* decay have been symbolized by the difference at these two wavelengths (Figure 3, panels B and D). This deconvolution procedure is validated by the fact that the Chl* signal decays to zero within 200 ns and stays stable until the end of the kinetics, *i.e*. 5 μs. This procedure also allowed us to normalize the data on the 440–460 signal at 2.5 ns, so as to compare the relative formations of ^3^Car* in either lutein- or violaxanthin reconstituted proteins. Kinetic data were fitted to a biexponential model (solid symbols in Figure 3B,D).

### Protein thermal stability

This was analyzed as described [21] by following the decrease of the 492 nm CD signal detected by a Jasco 600 spectropolarimeter at increasing temperatures, from 20 to 80°C (scan rate 1°C/minute and step 0.2°C). Protein thermal stability was measured as denaturation T°_1/2_, determined by inflection point of CD denaturation curves.

### Photobleaching assay

The kinetics of antennae photobleaching was measured as described [18] but with a higher light intensity of ca 6000 μmol m^-2^s^-1 ^and sample cooling at 10°C. Initial and maximal absorbance was 0.6.

### Determination of the sensitivity to photoxidative stress

Photoxidative stress was induced in whole plants by a strong light treatment at low temperature. Whole Arabidopsis plants were exposed to high light (1100 μmol photon m^-2 ^s^-1 ^with a photoperiod of 8 h) at low temperature (7°C/8°C, day/night air temperature), as described previously [66].

Photoinhibition of PSII was measured by chlorophyll fluorometry (F_v_/F_m _ratio) with a PAM-2000 fluorimeter (Walz). Photoxidative stress was measured by thermoluminometry with a custom-made apparatus that has been described [7]. The amplitude of the TL peak at 135°C was used as an index of lipid peroxidation.

## List of abbreviations

α(β)-DM, α(β)-dodecyl maltoside; A, antheraxanthin; Chl, chlorophyll; ^1^Chl*, chlorophyll excited singlet state; ^3^Chl*, chlorophyll excited triplet state; Car, carotenoid;^3^Car*, carotenoid excited triplet state; CD, circular dichroism; DCMU, 3-(3,4-dichlorophenyl)-1,1-dimethylurea; IEF, isoelectric focusing; lhc, light-harvesting complex; LHCII, major light harvetsing complex of photosystem II; L, lutein; N, neoxanthin; NPQ, non-photochemical quenching; ^1^O_2_*, singlet oxygen; ; PSII (I), photosystem II (I); qE, ΔpH-dependent portion of non-photochemical quenching; ROS, reactive oxygen species; V, violaxanthin; VDE, violaxanthin de-epoxidse; Z, zeaxanthin.

## Authors' contributions

LD carried out the crossing to obtain double mutant *npq1 lut2.1*, the characterization of either whole pants and isolated Lhc complexes, the photoxidative treatments and stress measurements. CL carried out the isolation of *lut2.1 *mutant. JA was involved in measurements of time-resolved spectroscopy for carotenoid triplet formation. MH carried out thermoluminescence and photosynthetic O_2 _evolution measurements. RB e GG conceived of the study, and participated in its design and coordination and helped to draft the manuscript. All authors read and approved the final manuscript.

## Supplementary Material

Additional file 1 Pigment composition of leaf tissue from WT and mutant genotypes, both dark adapted, and after light stress (1200 μmol m-2 s-1, 20' at RT). Click here for file

Additional file 2 Photosynthetic parameters Fo, Fm, Fv/Fm and T1/2 measured on WT and lut2.1 leaves. Click here for file

Additional file 6 Kinetics of NPQ. Click here for file

Additional file 8 Fitting of the Soret region of LHCII 1-T spectra from WT and *lut2.1*. Click here for file

Additional file 3 Pigment composition and stability to heat denaturation of fractions obtained by preparative IEF of monomeric Lhcb isolated from WT and *lut2.1*. Click here for file

Additional file 7 Thermal stability of Lhcb proteins isolated from WT and *lut2.1 *thylakoids. Click here for file

Additional file 9 Flat bed isoelectric focusing fractionation. Click here for file

Additional file 4 Leaf pigment composition of 3-weeks-old WT and *lut2.1 *plants grown for three additional weeks LL (120 μmol m^-2 ^s^-1^) or HL (1400 μmol m^-2 ^s^-1^) conditions. Click here for file

Additional file 5 Measurements of Fv/Fm ratio and chlorophyll content of WT and mutant leaves during growth at low temperature and different light conditions. Click here for file

## Figures and Tables

**Table 1:** Emerson enhancement of oxygen evolution measured on WT and *lut2.1 *leaves.

**genotypes**	**State II**	**State I**
**WT**	5.7 ± 0.9	28.8 ± 2.8
***lut2.1***	15.3 ± 3.3	19.1 ± 4.8

O_2 _evolution was measured with the photoacoustic method (see Experimental Procedures for details). The Emerson enhancement was determined by comparing state I (obtained after 10 min. illumination with far-red light) to state II (obtained after 10 min. illumination with blue-green light).

**Table 2:** Pigment composition of monomeric Lhcb (from WT and *lut2.1*) and trimeric LHCII (from WT).

	**Chl a/b**	**Chl/Car**	**Neo**	**Viola**	**Anthera**	**Lute**	**Zea**	**beta-Car**
WT – band 2	1.8 ± 0.1	3.6 ± 0.1	5.9 ± 0.3	2.3 ± 0.1	nd	10.5 ± 0.4	nd	nd
WT – band 3	1.5 ± 0.1	3.8 ± 0.1	5.9 ± 0.2	1.6 ± 0.1	nd	10.7 ± 0.4	nd	nd
*lut2.1 *– band 2	1.5 ± 0.1	4.1 ± 0.1	9.8 ± 0.1	10.5 ± 0.1	3.2 ± 0.1	nd	1.1 ± 0.1	nd

Bands 2 and 3 were isolated from solubilized thylakoid membranes by sucrose gradient ultracentrifugation. Data are normalized to 100 Chl a+b, and they are expressed as mean ± SD, n = 3. nd, not detected.

**Table 3:** Xanthophyll spectral forms and efficiency of energy transfer to Chl a in LHCII monomeric preparations purified by non-denaturing IEF from WT and *lut2.1 *thylakoids.

		Site			
		
		L1	L2		N1
LHCII WT	Spectral form	Lutein1 (489 nm)	Lutein2 (495 nm)	Viola1 (492 nm)	Neoxanthin (486.5 nm)
	Efficiency	81.0%	79.0%		60.0%
LHCII *lut2.1*	Spectral form	Viola2 (505 nm)		Viola1 (493.5 nm)	Neoxanthin (486.5 nm)
	Efficiency	93.8%		79.0%	60.0%

Spectral deconvolution analysis and calculation of energy transfer efficiency were as in Croce et al.,, 1999 [18]. The data, normalized to the WT, are relative to a 100% Chl a-to-Chl a ET efficiency. The error in the ET efficiency was <4%, with the exception of Viola1 in WT (>10%). Xanthophyll absorption maxima in ethanol are 477.2, 472.8 and 468.4 nm, respectively, for violaxanthin, lutein and neoxanthin. Binding to sites L2 and L1 shifts violaxanthin absorption from 477.2 to 492 and 505 nm respectively; lutein is shifted from 472.8 to 489 and 495 nm, respectively. Binding to site N1 shifts neoxanthin from 468.4 to 486.5 nm.
