# Supplementary material for: Lutein is needed for efficient chlorophyll triplet quenching in the major LHCII antenna complex of higher plants and effective photoprotection in vivo under strong light
Source: BMC Plant Biol. 2006 Dec 27;6:32. doi: 10.1186/1471-2229-6-32 (PMC1769499; doi:10.1186/1471-2229-6-32)
Supplement: Additional file 1 — Pigment composition of leaf tissue from WT and mutant genotypes, both dark adapted, and after light stress (1200 μmol m-2 s-1, 20' at RT). [file 1471-2229-6-32-S1.pdf]

Table a1. Pigment composition of leaf tissue from WT and mutant genotypes, both dark adapted, and after light stress (1200 µmol m^{-2} s^{-1}, 20' at RT).

Data are normalized to 100 Chl *a* +*b* molecules, and are expressed as mean ± SD, n=3. See Methods for details. Abbreviations: beta, β-carotene. nd, not detected.

	Genotypes	Chl a/b	Chl/Car	Neo	Viola	Anthera	Lute	Zea	beta-Car
dark-adapted leaves	**WT**	3.1 ± 0.1	3.4 ± 0.1	5.7 ± 0.6	3.3 ± 0.1	nd	11.8 ± 0.1	nd	8.7 ± 0.4
	lut2.1	3.6 ± 0.1	3.5 ± 0.1	5.3 ± 0.5	9.8 ± 0.8	0.9 ± 0.1	nd	0.3 ± 0.1	12.2 ± 0.1
	npq1	3.1 ± 0.1	4.5 ± 0.2	4.4 ± 0.3	2.6 ± 0.3	nd	9.6 ± 0.9	nd	8.2 ± 0.1
	npq1 lut2.1	3.6 ± 0.1	3.6 ± 0.1	4.9 ± 0.1	9.2 ± 0.3	0.8 ± 0.3	nd	0.4 ± 0.1	12.8 ± 0.2
light-treated leaves (20', 1200 µmol $m^{-2}s^{-1}$)	**WT**	3.1 ± 0.1	3.4 ± 0.1	5.1 ± 0.3	1.4 ± 0.1	0.4 ± 0.1	12.2 ± 0.2	1.7 ± 0.1	8.9 ± 0.3
	lut2.1	3.5 ± 0.1	3.6 ± 0.1	4.3 ± 0.6	4.7 ± 0.1	1.3 ± 0.2	nd	5.1 ± 0.1	12.6 ± 0.2
	npq1	3.1 ± 0.1	4.3 ± 0.1	4.4 ± 0.3	2.6 ± 0.3	nd	9.7 ± 0.8	nd	8.1 ± 0.4
	npq1 lut2.1	3.6 ± 0.1	4.2 ± 0.1	5.0 ± 0.7	9.6 ± 0.1	0.9 ± 0.3	nd	0.4 ± 0.1	11.0 ± 0.9
