# Supplementary material for: Lutein is needed for efficient chlorophyll triplet quenching in the major LHCII antenna complex of higher plants and effective photoprotection in vivo under strong light
Source: BMC Plant Biol. 2006 Dec 27;6:32. doi: 10.1186/1471-2229-6-32 (PMC1769499; doi:10.1186/1471-2229-6-32)
Supplement: Additional file 2 — Photosynthetic parameters Fo, Fm, Fv/Fm and T1/2 measured on WT and lut2.1 leaves. [file 1471-2229-6-32-S2.pdf]

Table a2. Photosynthetic parameters F_o, F_m, F_v/F_m and $T_{1/2}$ measured on WT and *lut2.1* leaves.

Parameters were obtained from measurements on whole leaf, with a PAM-2000 fluorimeter and expressed as mean ± SD. The half time of the fluorescence rise ($T_{1/2}$) was measured in DCMU-infiltrated leaves, and was taken as a measure of the functional antenna size of PSII.

genotypes	F_o (a.u.)	F_m (a.u.)	F_v/F_m	$T_{1/2}$ (ms)
**WT**	10271 ± 199	48717 ± 2230	0.79 ± 0.06	65.2 ± 3.9
lut2.1	7370 ± 485	34886 ± 1869	0.79 ± 0.07	81.0 ± 7.5
