# Supplementary material for: Lutein is needed for efficient chlorophyll triplet quenching in the major LHCII antenna complex of higher plants and effective photoprotection in vivo under strong light
Source: BMC Plant Biol. 2006 Dec 27;6:32. doi: 10.1186/1471-2229-6-32 (PMC1769499; doi:10.1186/1471-2229-6-32)

Figure a1. Kinetics of NPQ. *In vivo* Non-Photochemical Quenching of chlorophyll fluorescence was induced by saturating actinic light (1200 μmol m^{-2} s^{-1}) on WT and mutant leaves. The values of NPQ were calculated as described in Experimental Procedures. Measures were repeated at least on 4 leaves.

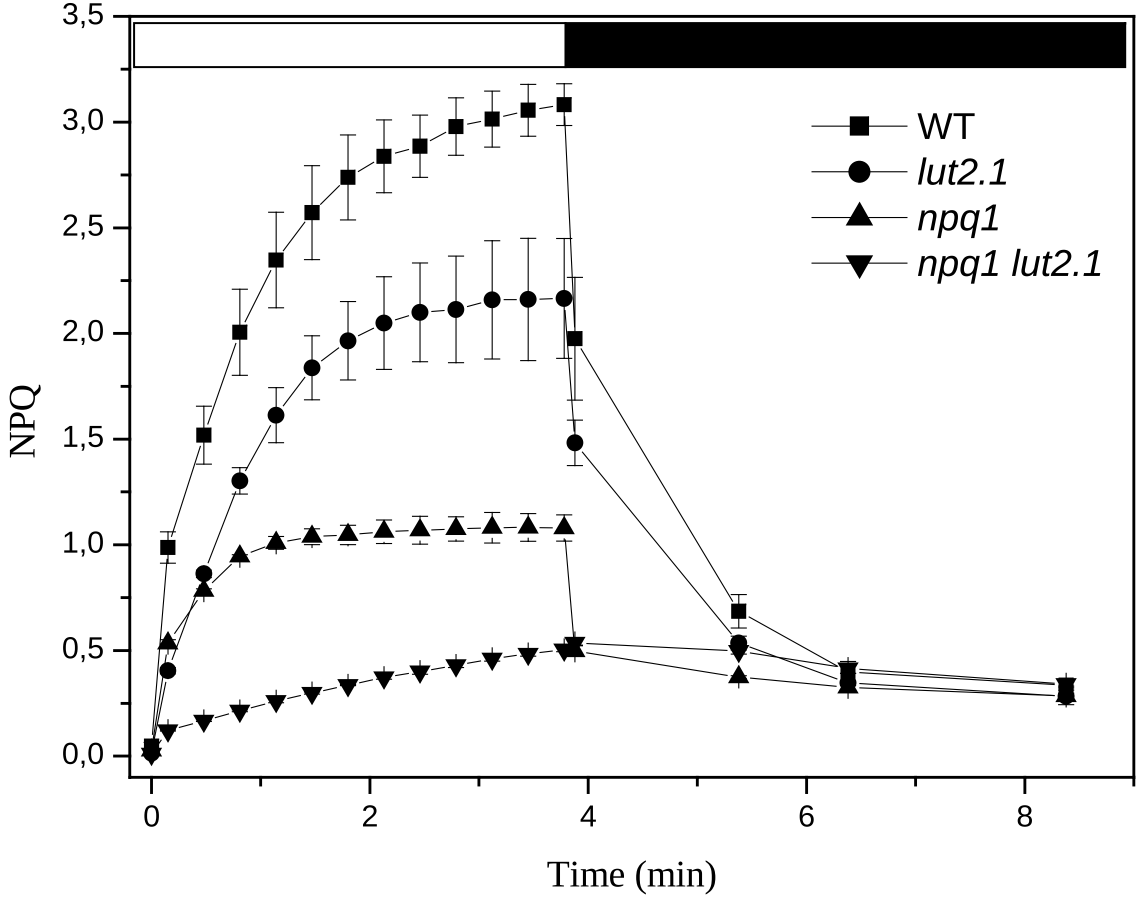

Supplement: Additional file 6 — Kinetics of NPQ. [file 1471-2229-6-32-S6.pdf]
