# Supplementary material for: Lutein is needed for efficient chlorophyll triplet quenching in the major LHCII antenna complex of higher plants and effective photoprotection in vivo under strong light
Source: BMC Plant Biol. 2006 Dec 27;6:32. doi: 10.1186/1471-2229-6-32 (PMC1769499; doi:10.1186/1471-2229-6-32)

Figure a3. Fitting of the Soret region of LHCII absorption spectra from WT and *lut2.1*. Description of the Soret region were performed on the absorption (A, C) and excitation (B, D) spectra of purified, monomeric LHCII form WT (A, B) and *lut2.1* (C, D). Spectra of both complexes (solid line) are compared with the reconstituted spectra obtained as a sum of the bands of single pigments (dotted line). The spectra of individual pigments are also shown. Spectra were recorded at RT.

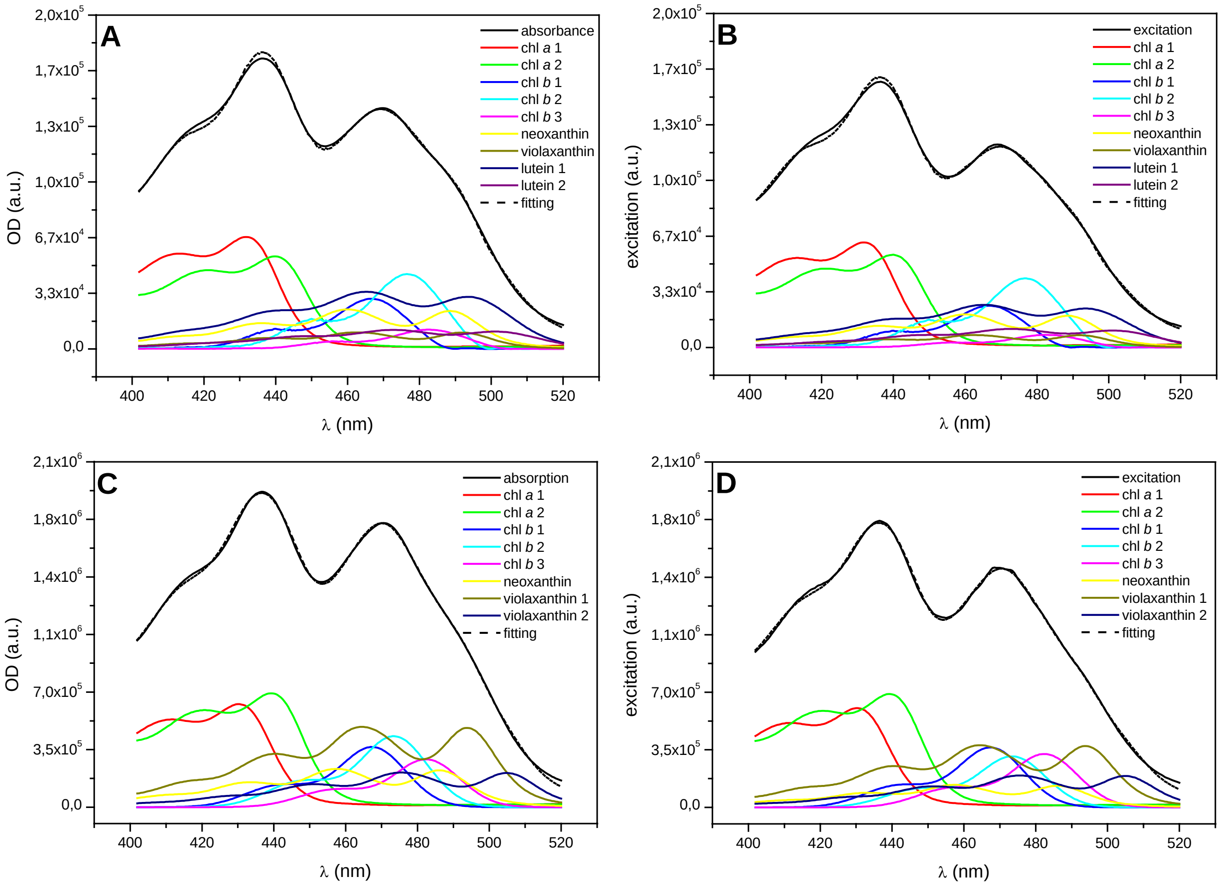

Supplement: Additional file 8 — Fitting of the Soret region of LHCII 1-T spectra from WT and lut2.1. [file 1471-2229-6-32-S8.pdf]
