# Supplementary material for: Lutein is needed for efficient chlorophyll triplet quenching in the major LHCII antenna complex of higher plants and effective photoprotection in vivo under strong light
Source: BMC Plant Biol. 2006 Dec 27;6:32. doi: 10.1186/1471-2229-6-32 (PMC1769499; doi:10.1186/1471-2229-6-32)
Supplement: Additional file 3 — Pigment composition and stability to heat denaturation of fractions obtained by preparative IEF of monomeric Lhcb isolated from WT and lut2.1. [file 1471-2229-6-32-S3.pdf]

Table a3. Pigment composition and stability to heat denaturation of fractions obtained by preparative IEF of monomeric Lhcb isolated from WT and *lut2.1* .

Data are normalized to 100 Chl *a* +*b* molecules, and are expressed as mean ± SD, n=3. Thermal stability was measured by heat denaturation dependence of the major CD signal at 492 nm. nd, not detected. See Experimental Procedures for details.

fractions	Chl *a* /*b*	Chl/Car	Neo	Viola	Anthera	Lute	Zea	stability (°C)
**WT - fr. 1**	1,4 ± 0,1	4,5 ± 0,1	5,9 ± 0,1	2,7 ± 0,1	nd	13,7 ± 0,1	nd	75,8 ± 0,8
***lut2.1* - fr. 1**	1,0 ± 0,1	4,7 ± 0,1	8,1 ± 0,1	9,8 ± 0,1	2,7 ± 0,1	nd	0,9 ± 0,1	74,9 ± 1,0
**WT - fr. 2**	1,3 ± 0,1	4,4 ± 0,1	5,8 ± 0,1	3,4 ± 0,2	nd	13,6 ± 0,3	nd	75,7 ± 0,6
***lut2.1* - fr. 2**	1,1 ± 0,1	4,5 ± 0,1	8,1 ± 0,1	10,0 ± 0,2	2,9 ± 0,2	nd	1,1 ± 0,1	74,6 ± 0,8
**WT - fr. 3**	1,5 ± 0,1	5,2 ± 0,1	4,1 ± 0,1	5,2 ± 0,1	nd	9,9 ± 0,1	nd	63,3 ± 0,2
***lut2.1* - fr. 3**	1,6 ± 0,1	4,9 ± 0,1	5,3 ± 0,1	10,4 ± 0,2	3,2 ± 0,1	nd	1,3 ± 0,1	65,8 ± 0,2
**WT - fr. 4**	1,8 ± 0,1	5,4 ± 0,1	3,8 ± 0,1	3,8 ± 0,1	nd	10,9 ± 0,1	nd	59,1 ± 0,1
***lut2.1* - fr. 4**	1,8 ± 0,1	5,2 ± 0,1	5,0 ± 0,1	10,0 ± 0,3	3,3± 0,1	nd	1,1 ± 0,1	58,9 ± 0,2
**WT - fr. 5**	2,0 ± 0,1	6,6 ± 1,5	5,7 ± 0,2	2,2 ± 0,1	nd	7,2 ± 0,3	nd	60,6 ± 0,1
***lut2.1* - fr. 5**	1,9 ± 0,1	5,2 ± 0,1	5,5 ± 0,2	7,6 ± 0,1	4,3 ± 0,2	nd	1,9 ± 0,1	60,6 ± 0,2
