# Supplementary material for: Lutein is needed for efficient chlorophyll triplet quenching in the major LHCII antenna complex of higher plants and effective photoprotection in vivo under strong light
Source: BMC Plant Biol. 2006 Dec 27;6:32. doi: 10.1186/1471-2229-6-32 (PMC1769499; doi:10.1186/1471-2229-6-32)

Figure a2. **Thermal stability of Lhcb proteins isolated from WT and *lut2.1* thylakoids.** Thermal stability of minor antennae, monomeric and trimeric LHCII were measured by heat-denaturation dependence of the (-) CD signal at 492 nm. Analyses were performed on (A) monomeric Lhcb from WT, (B) trimeric LHCII from WT, and (C) monomeric Lhcb from *lut2.1*. For each curve, temperatures corresponding to inflection points are indicated.

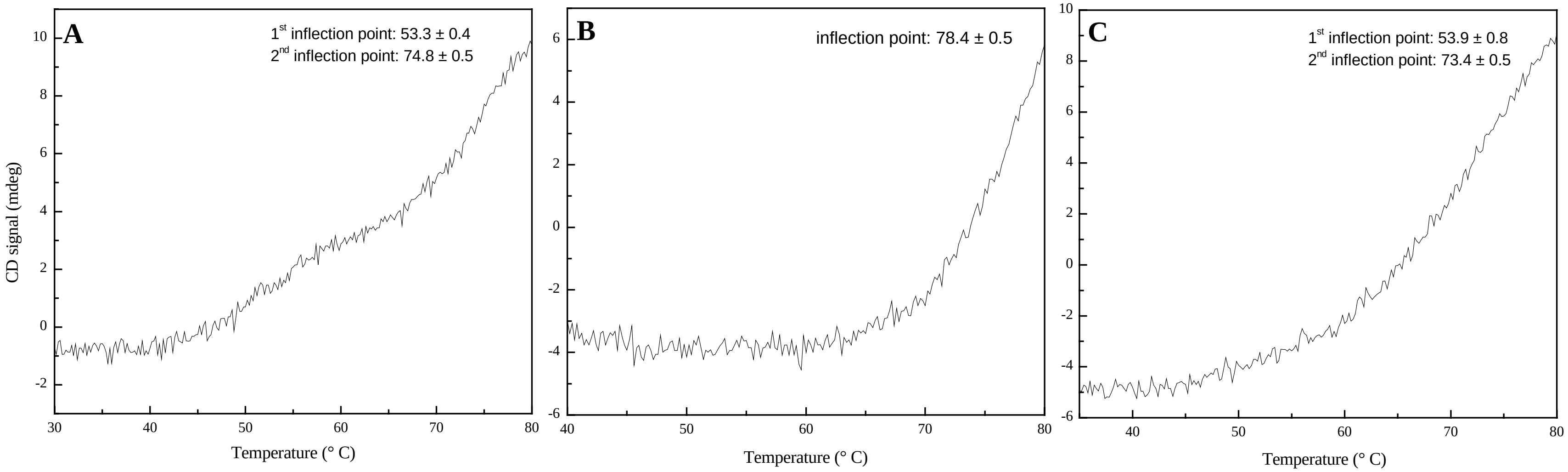

Supplement: Additional file 7 — Thermal stability of Lhcb proteins isolated from WT and lut2.1 thylakoids. [file 1471-2229-6-32-S7.pdf]
