# Supplementary material for: Lutein is needed for efficient chlorophyll triplet quenching in the major LHCII antenna complex of higher plants and effective photoprotection in vivo under strong light
Source: BMC Plant Biol. 2006 Dec 27;6:32. doi: 10.1186/1471-2229-6-32 (PMC1769499; doi:10.1186/1471-2229-6-32)

Figure a4. Flat bed isoelectric focusing fractionation. (A) Purification of monomeric Lhcb by preparative, flat-bed isoelectric focusing, isolated from WT and *lut2.1* solubilized thylakoid membranes by sucrose gradient ultracentrifugation. (B) Tris-Tricine SDS-PAGE analyses of fractions harvested from IEF. Protein composition of each fraction is indicated. After the run, gel was stained with Coomassie blue. See Experimental Procedures for details. Figure abbreviations: Fr, fraction; Thy, thylakoids; MW, molecular weight marker.

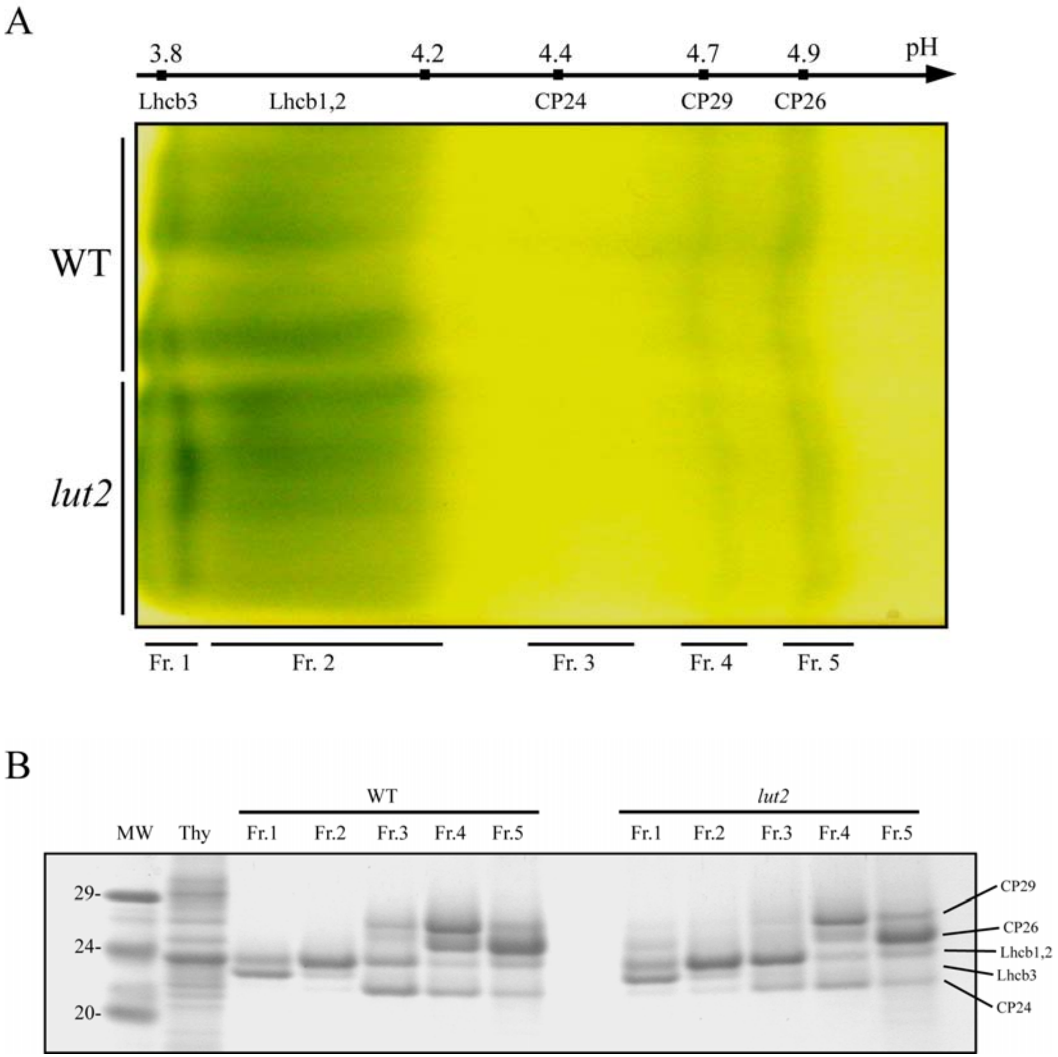

Supplement: Additional file 9 — Flat bed isoelectric focusing fractionation. [file 1471-2229-6-32-S9.pdf]
