# Supplementary material for: Lutein is needed for efficient chlorophyll triplet quenching in the major LHCII antenna complex of higher plants and effective photoprotection in vivo under strong light
Source: BMC Plant Biol. 2006 Dec 27;6:32. doi: 10.1186/1471-2229-6-32 (PMC1769499; doi:10.1186/1471-2229-6-32)
Supplement: Additional file 4 — Leaf pigment composition of 3-weeks-old WT and lut2.1 plants grown for three additional weeks LL (120 μmol m-2 s-1) or HL (1400 μmol m-2 s-1) conditions. [file 1471-2229-6-32-S4.pdf]

Table a4. Leaf pigment composition of 3-weeks-old WT and *lut2.1* plants grown for three additional weeks LL (120 µmol m^{-2} s^{-1}) or HL (1400 µmol m^{-2} s^{-1}) conditions.

Data are normalized to 100 Chl *a* +*b* molecules. Abbreviations: beta, β-carotene; nd, not detected. Data are expressed as mean ± SD, n=3.

	Chl *a/b*	Chl/Car	Neo	Viola	Anthera	Lute	Zea	beta-Car
**WT - 3 weeks HL**	3.5 ± 0.3	1.3 ± 0.1	14,6 ± 1,4	9,2 ± 0,4	0,7 ± 0,2	31,6 ± 1,6	0,5 ± 0,2	19,1 ± 1,2
**WT - 3 weeks LL**	3.1 ± 0.1	3.7 ± 0.1	6,9 ± 0,5	4,0 ± 0,7	nd	12,3 ± 0,5	nd	4,0 ± 0,3
***lut2.1* - 3 weeks HL**	4.0 ± 0.2	1.0 ± 0.2	17,8 ± 0,6	29,9 ± 0,8	7,5 ± 3,6	nd	6,9 ± 1,9	35,5 ± 6,1
***lut2.1* - 3 weeks LL**	3.5 ± 0.1	3.5 ± 0.1	8,2 ± 0,5	11,5 ± 0,4	2,0 ± 0,3	nd	1,0 ± 0,2	5,9 ± 0,1
