# Supplementary material for: Lutein is needed for efficient chlorophyll triplet quenching in the major LHCII antenna complex of higher plants and effective photoprotection in vivo under strong light
Source: BMC Plant Biol. 2006 Dec 27;6:32. doi: 10.1186/1471-2229-6-32 (PMC1769499; doi:10.1186/1471-2229-6-32)
Supplement: Additional file 5 — Measurements of Fv/Fm ratio and chlorophyll content of WT and mutant leaves during growth at low temperature and different light conditions. [file 1471-2229-6-32-S5.pdf]

Table a5. Measurements of F_v/F_m ratio and chlorophyll content of WT and mutant leaves during growth at low temperature and different light conditions.

F_v/F_m ratio was recorded as index of photoinhibition of PSII. Chlorophyll content is expressed as mass of pigments (µg) per unit leaf surface (cm^2). Data refer to plants immediately prior to transfer at 4°C (t_o) and after 1, 2 and 3 weeks of growth in HL (800 µmol m^{-2} s^{-1}, 4°C) or LL (20 µmol m^{-2} s^{-1}, 4°C). Data are expressed as mean ± SD, n=6.

		**F_v/F_m ratio**				**Chlorophyll content (µg/cm^2)**			
		**t_o**	**1 week**	**2 weeks**	**3 weeks**	**t_o**	**1 week**	**2 weeks**	**3 weeks**
**HL**	**WT**	0.807 ± 0.014	0.723 ± 0.002	0.767 ± 0.003	0.764 ± 0.024	17.9 ± 1.9	14.7 ± 0.6	21.2 ± 1.3	22.3 ± 1.4
	npq1	0.820 ± 0.007	0.602 ± 0.021	0.725 ± 0.054	0.766 ± 0.018	18.5 ± 1.1	16.2 ± 2.4	28.0 ± 5.8	26.2 ± 5.4
	lut2.1	0.815 ± 0.008	0.669 ± 0.026	0.760 ± 0.040	0.778 ± 0.009	18.7 ± 0.4	16.9 ± 2.1	23.6 ± 2.7	24.8 ± 2.8
	npq1 lut2.1	0.800 ± 0.01	0.640 ± 0.027	0.781 ± 0.007	0.774 ± 0.006	20.7 ± 1.6	9.06 ± 1.4	19.4 ± 3.0	15.5 ± 2.4
**LL**	**WT**	0.807 ± 0.014	0.795 ± 0.006	0.798 ± 0.013	0.797 ± 0.011	17.9 ± 1.9	20.5 ± 1.2	21.0 ± 2.0	23.3 ± 2.1
	npq1	0.820 ± 0.007	0.791 ± 0.004	0.785 ± 0.014	0.785 ± 0.007	18.5 ± 1.1	22.2 ± 1.2	31.2 ± 3.55	28.7 ± 3.27
	lut2.1	0.815 ± 0.008	0.807 ± 0.020	0.810 ± 0.017	0.817 ± 0.014	18.7 ± 3.9	19.1 ± 1.6	23.7 ± 5.3	22.3 ± 3.6
	npq1 lut2.1	0.800 ± 0.010	0.800 ± 0.016	0.829 ± 0.021	0.791 ± 0.010	20.7 ± 1.6	19.4 ± 1.3	25.3 ± 2.2	24.1 ± 2.1
